# Excretion, Metabolism, and Tissue Distribution of *Gelsemium elegans* (*Gardn. & Champ.*) *Benth* in Pigs

**DOI:** 10.3390/molecules27082605

**Published:** 2022-04-18

**Authors:** Xiao Ma, Zi-Yuan Wang, Meng-Ting Zuo, Kun Yang, Zhi-Liang Sun, Yong Wu, Zhao-Ying Liu

**Affiliations:** 1 College of Veterinary Medicine, Hunan Agricultural University, 1 Nongda Rd, District Furong, Changsha 410128, China; maxiao4489@163.com (X.M.); 15874806252@163.com (Z.-Y.W.); zuomengting@aliyun.com (M.-T.Z.); yangkun409@163.com (K.Y.); sunzhiliang1965@aliyun.com (Z.-L.S.); 2 Hunan Engineering Technology Research Center of Veterinary Drugs, Hunan Agricultural University, 1 Nongda Rd, District Furong, Changsha 410128, China; 3 Hunan Canzoho Biological Technology Co., Ltd., 321 Kangning Road, District Economic and Technological Development, Liuyang 410329, China

**Keywords:** absorption, excretion, tissue distribution, metabolic profile, *Gelsemium*

## Abstract

*Gelsemium elegans* (*Gardn. & Champ.*) *Benth* is a toxic flowering plant in the family Loganiaceae used to treat skin diseases, neuralgia and acute pain. The high toxicity of *G. elegans* restricts its development and clinical applications, but in veterinary applications, *G. elegans* has been fed to pigs as a feed additive without poisoning. However, until now, the in vivo processes of the multiple components of *G. elegans* have not been studied. This study investigates the excretion, metabolism and tissue distribution of the multiple components of *G. elegans* after feeding it to pigs in medicated feed. Pigs were fed 2% *G. elegans* powder in feed for 45 days. The plasma, urine, bile, feces and tissues (heart, liver, lung, spleen, brain, spinal cord, adrenal gland, testis, thigh muscle, abdominal muscle and back muscle) were collected 6 h after the last feeding and analyzed using high-performance liquid chromatography coupled to quadrupole time-of-flight mass spectrometry. Five natural products in plasma, twelve natural products and five metabolites in urine, and three natural products in feces were characterized, suggesting that multiple components from *G. elegans* were excreted in the urine. However, ten natural products and four metabolites were detected in bile samples, which suggested that *G. elegans* is involved in enterohepatic circulation in pigs. A total of seven of these metabolites were characterized, and four metabolites were glucuronidated metabolites. Ten natural products and six metabolites were detected in the tissues, which indicates that *G. elegans* is widely distributed in tissues and can cross the blood-brain barrier. Among the characterized compounds, a highly toxic gelsedine-type alkaloid from *G. elegans* was the main compound detected in all biological samples. This is the first study of the excretion, metabolism and tissue distribution of multiple components from *G. elegans* in pigs. These data can provide an important reference to explain the efficacy and toxicity of *G. elegans*. Additionally, the results of the tissue distribution of *G. elegans* are of great value for further residue depletion studies and safety evaluations of products of animals fed *G. elegans*.

## 1. Introduction

*Gelsemium* is a genus of flowering plants in the Loganiaceae family [1]. There are four species in this genus: the Asian species, *Gelsemium elegans* (*Gardn. & Champ.*) *Benth* (*G. elegans*) and *G. rankii Small* and two North American species (*Gelsemium sempervirens* (L.) *J.St.-Hil.* and *Gelsemium rankinii Small*) [2]. The use of *Gelsemium sempervirens* traces back to the 19th century as a homeopathic agent to reduce anticipatory anxiety [3]. Pharmacological reports of *Gelsemium rankinii Small* are scarce because it is a rare species from the southeastern United States [4]. In China, *G. elegans* has been reported for its analgesic and anti-inflammatory effects and has been used to treat skin diseases, neuralgia and acute pain [1]. However, the high toxicity of *G. elegans* is the main limiting factor for its clinical applications. Generally, limb weakness, vomiting, arrhythmia, coma and other symptoms will occur after intoxication, and severe poisoning could lead to death [5]. However, *G. elegans* has growth-promoting effects in pigs in traditional Chinese medicine, so *G. elegans* could be added to feed [6]. However, this will cause *G. elegans* residue in products of animal origin and pose a threat to the safety of consumers, so it is necessary to study the excretion, metabolism and tissue distribution of *G. elegans*.

There are many compounds in *G. elegans*. Up to now, a total of 121 alkaloids, 25 iridoids, and several other compounds from a wide spectrum of secondary metabolite classes have been found in *G. elegans*. Based on the chemical structures of the alkaloids, they have been classified into the following six types: gelsedine-type, gelsemine-type, humantenine-type, koumine-type, sarpagine-type and yohimbane-type [7,8,9]. Among them, the contents of koumine and gelsemine are high, and these types can be rapidly absorbed and widely distributed in tissues and can pass through the blood-brain barrier in rats. Moreover, their toxicity is low, with LD_50_ values of 99 mg/kg and 56.2 mg/kg [1], respectively. The toxicity of gelsenicine is high, with an LD50 of 0.185 mg/kg [1], and it also has the characteristics of fast absorption, wide distribution, and the ability to cross the blood-brain barrier in rats and mice [10]. A previous study characterized CYP3A4/5 as the main metabolic enzyme of *G. elegans* alkaloids, which can reduce the toxicity of *G. elegans* alkaloids through metabolism [11]. In addition, our previous studies investigated the pharmacokinetics of a *G. elegans* extract in pigs and found that *G. elegans* alkaloids were characterized by fast absorption and wide distribution, which are similar characteristics to single alkaloids in rats and mice [12]. However, the metabolism and tissue distribution of *G. elegans* in pigs have not been studied.

Significant differences have been observed between species with respect to the toxicity of *G. elegans*. According to the existing studies, it has been speculated that the differences in *G. elegans* toxicity may be closely related to metabolism [11,13]. Many studies have found differences in the metabolism of koumine and gelsemine in liver microsomes of pigs and rats in vitro. However, these previous studies have all been based on the metabolism of single alkaloids in vitro, which cannot completely reflect the metabolism and distribution of multiple components of *G. elegans* in vivo. Only one study by our team has reported the metabolic profile of multiple components of *G. elegans* in goats. Therefore, it is important to investigate the in vivo excretion, tissue distribution, and metabolic profile of *G. elegans* in pigs.

The present study characterized the multiple components of *G. elegans* in pig plasma, urine, bile, feces, and tissues by using high-performance liquid chromatography coupled to quadrupole time-of-flight mass spectrometry (HPLC/QqTOF-MS). This study explored the excretion, metabolism and tissue distribution of *G. elegans* in pigs. These results may play an important role in explaining the toxicological and pharmacological effects of *G. elegans*. In addition, the tissue distribution data may provide a reference for further study on the elimination of tissue residues of *G. elegans* and the safety of products of animal origin, ensuring the safety of consumers.

## 2. Results

**Figure 1:**
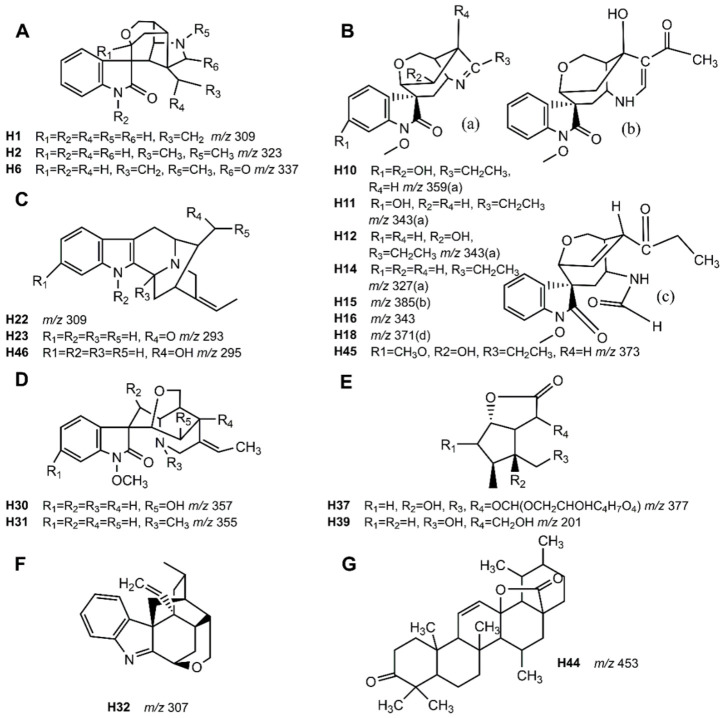
Main structures of the natural products identified in *G. elegans*: Gelsemine-type alkaloids (**A**), gelsedine-type alkaloids (**B**), sarpagine-type alkaloids (**C**), humantenine-type alkaloids (**D**), iridoids (**E**), koumine-type alkaloids (**F**), and triterpenes (**G**).

Recently, metabolism studies of *G.* *elegans* in goats were reported by our team [14]. The current study’s naming and analysis strategies of the characterized natural products and metabolites were consistent with the previously reported publication. As a result, twenty natural products and seven metabolites were characterized in plasma, urine, bile, feces and tissues. Information on these compounds is shown in Table 1. The main structures of the natural products are shown in Figure 1.

### 2.1. Characterization of Compounds in Plasma

**Figure 2:**
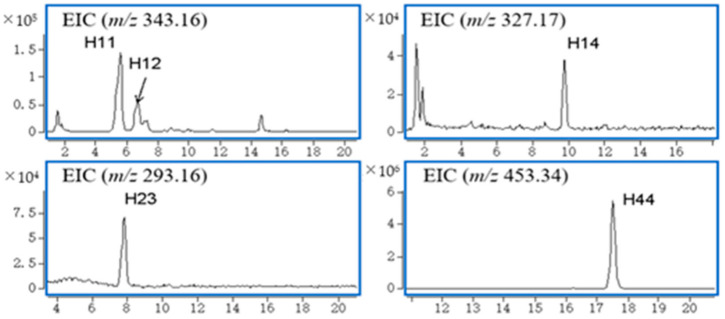
Accurate EICs of natural products and metabolites in plasma.

Five natural products from *G. elegans* were characterized in plasma samples, and the accurate EICs of these compounds are shown in Figure 2. No metabolites of the natural products were found in plasma samples.

These five natural products were gelsedine-type alkaloids (compounds **H11**, **H12**, **H14**), a sarpagine-type alkaloid (compound **H23**), and a triterpene (compound **H44**). These five natural products were not found in blank samples. All of them were characterized based on their characteristic accurate MS2 fragment (Table 1). **H12** and **H14** were unique to plasma and were not detected in bile, urine or fecal samples. Compound **H11** in the plasma sample was also detected in bile and urine samples. The MS2 spectrum of **H14** exhibited a product ion at *m*/*z* 296, formed by the loss of CH3O from *m*/*z* 327. The ion at *m*/*z* 296 could further lose CH3O to produce an ion at *m*/*z* 265. The product ion at *m*/*z* 225 was formed by the loss of C_3_H_4_ from *m*/*z* 265; after comparison with the reference data, **H14** was characterized as gelsenicine. The MS2 spectrum of **H11** generated product ions at *m*/*z* 312 and *m*/*z* 281, which were 16 Da higher than the *m*/*z* values of 296 and 265 of the product ions of gelsenicine, respectively. The product ion at *m*/*z* 108 was the same as that in gelsenicine, so **H11** is presumably 11-hydroxygelsenicine.

### 2.2. Characterization of Compounds in Urine

**Figure 3:**
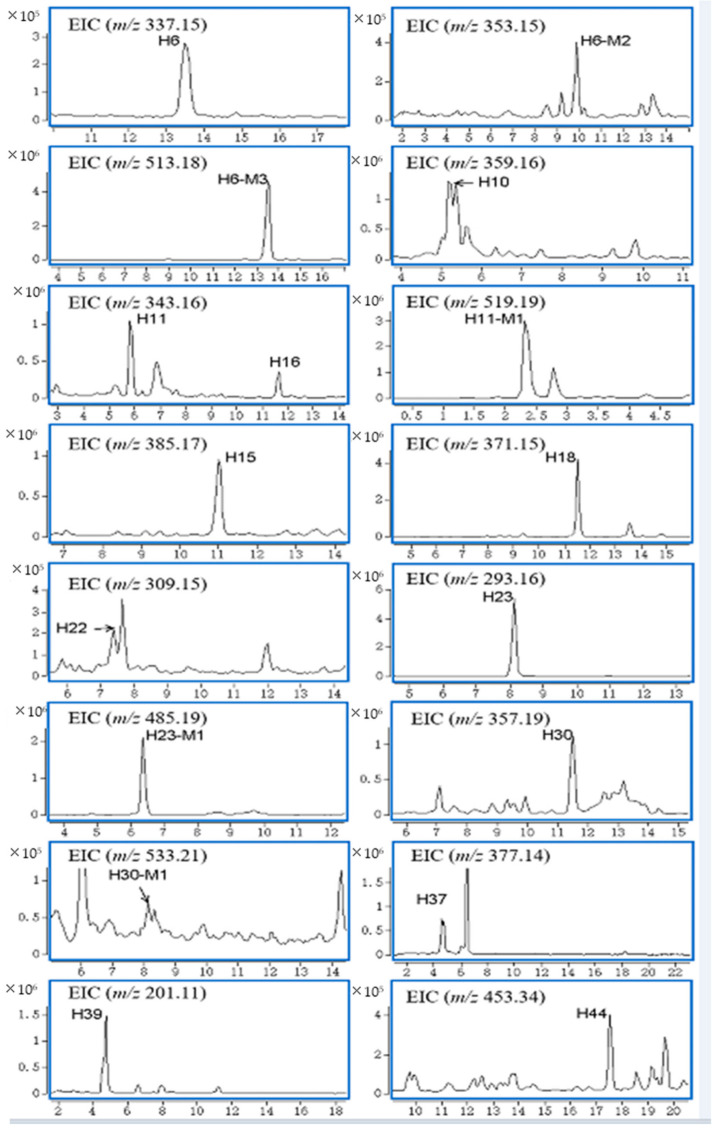
Accurate EICs of natural products and metabolites in urine.

Compared with the blank group, in all the biological samples of the experimental group, the highest number of compounds was detected in the urine samples, which shows that the metabolism of *G. elegans* is fast. Twelve natural products and five metabolites were characterized, and their EICs are shown in Figure 3.

#### 2.2.1. Gelsemine-Type Alkaloids (**H6**) and Its Metabolic Products

Gelsemine-type natural products (**H6**) and two related metabolites (**H6-M2** and **H6-M3**) were detected and identified. H6 was characterized as either gelsemine or 21-oxogelsemine based on comparing the data with the reference compounds. Metabolite **H6-M2** was characterized as an oxidation metabolite of **H6**. Additionally, a phase II metabolite conjugated with glucuronic acid was detected and named **H6-M3**.

The retention time of **H6** is 13.469 min, and the [M + H]^+^ was observed at *m*/*z* 337.1565. First, the MS2 spectrum of compound **H6** generated a product ion at *m*/*z* 236, formed by the loss of C_5_H_9_O_2_ from *m*/*z* 337, and the product ion at *m*/*z* 204 was due to the loss of C_8_H_7_NO from *m*/*z* 337. Then, the product ion at *m*/*z* 77 may have been formed by the loss of C_6_H_4_ from *m*/*z* 236. Therefore, we speculate that compound **H6** is 21-oxogelsemine.

Metabolite **H6-M2** presented an *m*/*z* of 353, which was 16 Da greater than that of natural product **H6**. The product ion at *m*/*z* 222 of **H6-M2** was also 16 Da higher than the product ion at *m*/*z* 206 of **H6**, suggesting that **H6-M2** is an oxidation metabolite of **H6**. Glucuronide conjugates could produce the [M + H − 176]^+^ fragment in the MS2 spectrum. The ion produced by the loss of C_6_H_8_O_6_ from metabolite **H6-M3** was the same as the *m*/*z* of compound **H6**. Therefore, metabolite **H6-M3** was characterized as a phase II glucuronic acid conjugated metabolite of compound **H6**.

#### 2.2.2. Gelsedine-Type Alkaloids (**H10**, **H11**, **H15**, **H16**, **H18**) and Their Metabolic Products

Five gelsedine-type natural products (compounds **H10**, **H11**, **H15**, **H16**, **H18**) and one related metabolite (**H11-M1**) were detected in urine samples. Compounds **H10**, **H11**, **H15**, **H16** and **H18** were characterized as 11,14-dihydroxygelsenicine, 11-hydroxygelsenicine, 15-hydroxyhumantenoxenine, hydroxygelsenicine and gelsemolenine B, respectively. Metabolite **H11-M1** may be a phase II metabolite conjugated with glucuronic acid.

The retention time of compound **H15** was 10.9 min, which was used as an example for the characterization of gelsedine-type compounds. The product ion at *m*/*z* 354 was formed by the loss of OCH_3_ from *m*/*z* 385, and further, the product ion at *m*/*z* 309 was formed by the loss of CHO_2_ from *m*/*z* 354. The product ion at *m*/*z* 134 may have been formed by loss of CH_4_NO from *m*/*z* 180. According to this fragmentation information, natural product **H15** was suggested to be 15-hydroxyhumantenoxenine.

Metabolite **H11-M1** may be a phase II glucuronic acid conjugated metabolite because the [M + H]^+^ of **H11-M1** was observed at *m*/*z* 519, 176 Da higher than the *m*/*z* of **H11**.

#### 2.2.3. Sarpagine-Type Alkaloids (**H23**)

There was only one compound (compound **H23**) belonging to the sarpagine type in bile samples, and no metabolites were found. In our previous study, the compound koumidine was found in the crude extract of *G. elegans*. The MS2 spectrum of **H23** was compared with the MS2 spectrum of koumidine, and the product ion at *m*/*z* 154 from **H23** was 2 Da lower than that of the product ion at *m*/*z* 156 of koumidine. Combined with the other product ions, **H23** was characterized as dehydrokoumidine.

#### 2.2.4. Humantenine-Type Alkaloids (**H30**) and Its Metabolic Products

Compound (**H30**) was classified as a humantenine-type alkaloid. The phase II metabolite of **H30** was characterized and named **H30-M1**.

The fragment ions of compound **H30** showed that the product ion at *m*/*z* 326 was formed by the loss of OCH_3_ from *m*/*z* 357. The product ion at *m*/*z* 311 was formed by the loss of CH_3_ from *m*/*z* 326, and the further loss of CH_2_ from the ion at *m*/*z* 311 followed to generate the *m*/*z* of 297. Compound **H30** was tentatively characterized as 14-hydroxyrankinidine.

Metabolite **H30-M1** displayed an [M + H]^+^ ion at *m*/*z* 533, which is consistent with the molecular formula of C_26_H_32_N_2_O_10_. The MS2 spectrum revealed that the [M + H]^+^ ion could further lose a glucuronic acid moiety (176 Da) to produce the fragment ion at *m*/*z* 357. The fragment ion at *m*/*z* 357 then fragmented into an ion at *m*/*z* 311, which was consistent with the fragmentation of **H30**. Therefore, we deduced that metabolite **H30-M1** was a glucuronidated metabolite of 14-hydroxyrankinidine.

#### 2.2.5. Non-Alkaloids (**H37**, **H39**, **H44**)

Two iridoid compounds (**H37** and **H39**) and one triterpene compound (**H44**) were found in urine samples. **H37** and **H39** were characterized as 9-hydroxysemperoside and gelsemiol. **H44** was characterized by triterpene 3-keto-urs-11-en-13β(28)-olidevia database matching.

### 2.3. Characterization of Compounds in Bile

**Figure 4:**
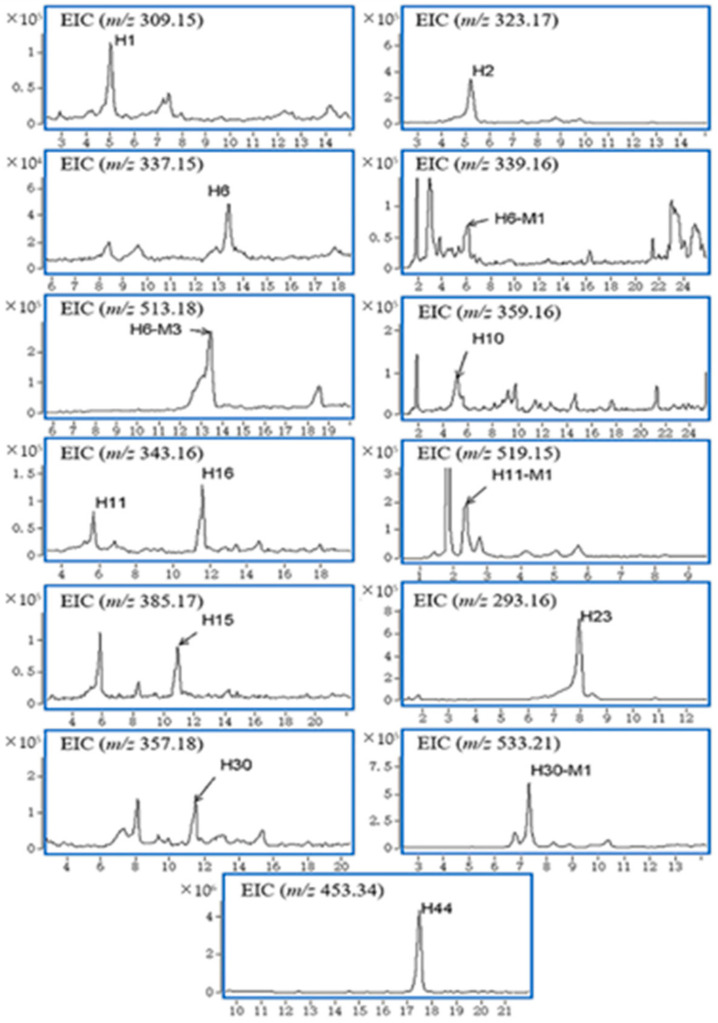
Accurate EICs of natural products and metabolites in bile.

A total of ten natural products and four metabolites were detected in bile samples. These ten natural products and four metabolites were not found in the blank samples. Accurate EICs of the natural products and metabolites in bile samples are shown in Figure 4.

Three of these natural products were determined to be gelsemine-type alkaloids (compounds **H1**, **H2**, **H6**), four were determined as gelsedine-type alkaloids (compounds **H10**, **H11**, **H15**, **H16**), and the other three were a sarpagine-type alkaloid (compound **H23**), a humantenine-type alkaloid (compound **H30**) and a triterpene (compound **H44**). Based on the MS2 data and accurate mass analysis, the structures of these compounds were characterized. The protonated molecular ion ([M + H]^+^) of **H2** was observed at *m*/*z* 323.1750 (C_20_H_23_N_2_O_3_^+^, 1.26 ppm). As observed from the MS2 spectrum, the more abundant product ion at *m*/*z* 236.1076 was formed by the neutral losses of CH_2_O (30 Da) and C3H7N (57 Da). **H2** was characterized as gelsemine by comparison with the reference standard.

A total of four metabolites (**H6-M1**, **H6-M3**, **H11-M1**, **H30-M1**) were detected in the bile samples. H6-M3, H11-M1 and H30-M1 may be phase II glucuronic acid conjugated metabolites of compounds because their [M + H]^+^ values were observed at *m*/*z* 519, 513 and 533, which were all 176 Da greater than **H6**, **H11** and **H30**, respectively. When the natural products (**H6**, **H11**, **H30**) were combined with glucuronic acid, the water solubility increased, followed by entry into the bile and participation in enterohepatic circulation.

### 2.4. Compounds Identification in Feces

**Figure 5:**
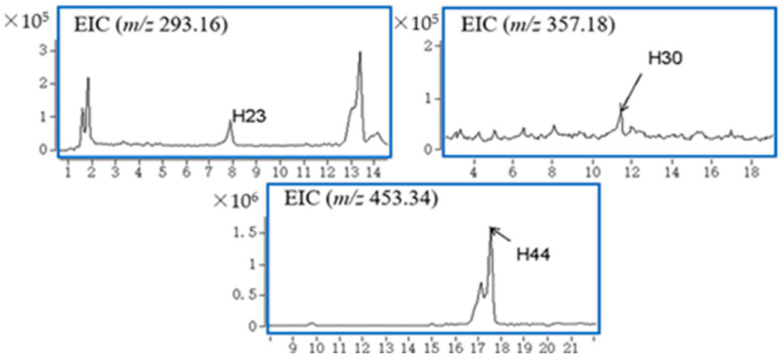
Accurate EICs of natural products and metabolites in feces.

Three compounds were tentatively identified via comparisons of the retention times and observed masses in feces samples, including one sarpagine-type alkaloids (**H23**), one humantanine-type alkaloids (**H30**), and a triterpene (**H44**). Only three natural products were detected in the feces of the experimental group, and no metabolites were detected. Many compounds were detected in bile but not in feces, suggesting that these compounds may have been reabsorbed. The accurate EICs of those compounds are shown in Figure 5.

### 2.5. Characterization of Compounds in Tissue

As a result, nine natural products were detected in the brain, which suggested that *G. elegans* could cross the blood-brain barrier. All compounds were a gelsemine-type alkaloid (**H2**), gelsedine-type alkaloids (**H11**, **H45**), sarpagine-type alkaloids (**H23**, **H46**), humantenine-type alkaloids (**H30**, **H31**), a koumine-type alkaloid (**H32**) and a triterpene (**H44**). **H46** and **H32** were selected as examples to characterize the fragmentation patterns for the other compounds. **H46** was characterized as koumidine, the major fragment ion at *m*/*z* 277 was due to the loss of H_2_O from the ion at *m*/*z* 295, and compared with the reference data, the structure was determined. Compound **H32** was characterized as koumine because the fragment ion at *m*/*z* 277 was formed by loss of CH_2_ from the ion at *m*/*z* 307 and the further loss of C_3_H_7_N to form an *m*/*z* of 220. Moreover, the ion at *m*/*z* 307 was formed by *m*/*z* 238 + *m*/*z* 70. Only three compounds were detected in the spinal cord, including gelsemine-type alkaloids (**H2**, **H45**) and a sarpagine-type alkaloid (**H23**), which were also detected in the brain. All compounds detected in the tissues are shown in Table 2.

A total of eight compounds were tentatively characterized in the heart, including **H2**, **H11**, **H14**, **H23**, **H30**, **H31**, **H32** and **H44**. Only compound **H23** was detected in the liver and adrenal gland, and their content was very low. Five natural products were characterized in the lung, including **H2**, **H14**, **H23**, **H46**, **H30**, and **H23-M2** were also detected. Compounds **H2**, **H11**, **H14**, **H23**, **H30**, **H32** and **H23-M2** were detected in the spleen and testis, compound **H46** was only found in the spleen, and **H31**, **H44** and **H30-M1** were only detected in the genitals.

Finally, compounds **H2**, **H11**, **H14**, **H23**, **H30** and **H32** were detected in the thigh, abdominal, and back muscle samples. However, **H31** was only found in abdominal and back muscles, and **H44** was only detected in back muscles.

Dehydrokoumidine (**H23**) was detected in all tissues, and its content in the lung was the highest. Gelsemine (**H2**) was also detected in most tissues except the lungs and adrenal gland and was highest in the brain. Two metabolites (**H23-M1**, **H30-M1**) were detected only in the lungs, spleen, and testis, indicating that most metabolites are highly polar and excreted through the kidneys and bile.

## 3. Discussion

Previously, there have been studies on the pharmacokinetics of koumine, gelsemine, and gelsenicine in rats or mice [10]. These three single alkaloids have rapid absorption and are widely distributed and rapidly eliminated. Subsequently, our study explored the multicomponent pharmacokinetics of *G. elegans* in pigs [15,16]. The results showed that *G. elegans* alkaloids were rapidly absorbed in pigs but eliminated more slowly (the value of T1/2 was 8 to 12 h) than in rats and mice. However, multiple components’ metabolism and tissue distribution have not yet been studied. This study is the first to study the metabolism and tissue distribution of multiple components of *G. elegans* in pigs. This study detected ten natural products and four metabolites in bile, but only three natural products were detected in feces. Therefore, it is speculated that the slow elimination of *G. elegans* in pigs may be because *G. elegans* is involved in enterohepatic circulation. In addition, this study did not detect as many compounds because the pharmacokinetics study used the HPLC/QqQ-MS method. Because the detected concentration was low in the pig biological samples, many compounds were not detected by HPLC/QqTOF-MS. Although it is not possible to detect all compounds, metabolism and distribution studies can be performed on the compounds with a high content, which are active substances with *G. elegans* effects.

**Figure 6:**
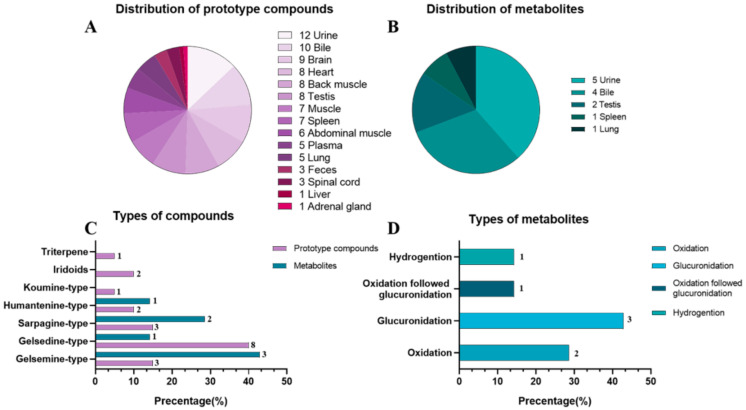
The number of natural products distributed in different biological samples (**A**). The number of metabolites distributed in different biological samples (**B**). The types of natural products and their metabolites in different biological samples (**C**). The types of metabolites formed by different metabolic pathways in different biological samples (**D**).

We used rat and pig liver S9 to investigate the in vitro metabolism of koumine and gelsemin [17,18]. The results show that the main metabolic pathways of koumine and gelsemine were dehydrogenation, hydrogenation, demethylation and oxidation, which was consistent with previous research results. However, these studies have not found phase II metabolites of these two alkaloids. Our previous study on goats used a single oral administration [14], but this study used long-term feeding. The method of administration was different, so the compounds detected in the biological samples of pigs were less than those observed in goats. In previous in vivo experiments in sheep, only plasma, urine and fecal samples were analyzed and determined, and a total of 44 absorbed natural products and 27 related metabolites were preliminarily characterized. Including gelsdine type, sarpagine type and gelsmeine type alkaloids are the compounds with the highest amount of metabolites. Most natural products are metabolized by glucuronidation and oxidation. In addition, hydrogenation, dehydrogenation and demethylation reactions also occur. However, this study analyzed and characterized the metabolites in plasma, urine, feces, bile and various tissues. This study first investigated the metabolic profile of multiple components of *G. elegans* in pigs. The metabolic pathways of 21-oxogelsemine (**H6**), 11-hydroxygelsenicine (**H11**), dehydrokoumidine (**H23**) and 14-hydroxyrankinidine (**H30**) in pigs are mainly reduced by hydrogenation, oxidation and glucuronidation. Many compounds were detected in bile in this study, especially phase II metabolites, indicating the existence of enterohepatic circulation in pigs, which may be the reason for the slow elimination of *G. elegans*. In addition, glucuronidation was the major metabolic pathway in pigs, accounting for 42.9%, as diagrammed in Figure 6D.

Compounds from *G. elegans* can be detected in all tissues, indicating that *G. elegans* is widely distributed in pigs. Figure 6A,B shows that most natural products were detected in the brain from all tissues, including koumine (**H32**) and gelsemine (**H2**). Some studies have shown that koumine and gelsemine have antianxiety and analgesic effects. These two alkaloids have low toxicity and potential for development, indicating that the overall administration of *G. elegans* was a multicomponent multitargeted mechanism. Moreover, it is shown in Figure 6C that the highly toxic alkaloids, the gelsedine type, were present in the highest concentrations in all biological samples, which may be the main reason for human poisoning after eating *G. elegans*. *G. elegans* was not toxic to pigs [19], possibly because of different pharmacokinetics and pharmacodynamics between the receptors and gelsedine-type alkaloids within the different species, but this needs further study. Additionally, gelsedine-type alkaloids have been characterized and detected in the lung, heart, and other tissues, indicating that *G. elegans* poisoning, respiratory failure, and rapid heartbeat may be caused by nerves’ direct and indirect effects. The study also found that compounds from *G. elegans* were distributed in the muscles and liver, which may threaten the safety of consumers of these animal products. Therefore, the withdrawal time required for *G. elegans* alkaloids to be eliminated from edible tissues needs further residue depletion studies.

In conclusion, high-performance liquid chromatography coupled to quadrupole time-of-flight mass spectrometry was used in this study to characterize multiple components of *G. elegans* in pig plasma, urine, feces, bile and different tissues. This is the first study to provide comprehensive information on the absorption, excretion, metabolism and tissue distribution of multiple components of *G. elegans* in pigs. These findings may help determine gelsedine-type alkaloids from *G. elegans* for further toxicity and residue studies, laying the foundation for further safety evaluations of products of animals fed *G. elegans* and promoting the development and utilization of *G. elegans* in livestock farming.

## 4. Materials and Methods

### 4.1. Chemicals and Reagents

Gelsemine (CAS#: 509-15-9, purity: 99.28%), koumine (CAS#: 1358-76-5, purity: 99.53%) and humantenmine (CAS#: 354-38-9, purity: 99.84%) were purchased from Shanghai Kang Biao Chemicals Co., Ltd. (Minhang District, Shanghai, China). Methanol, acetonitrile and formic acid were obtained from Merck Chemicals Co. (Darmstadt, Germany). Water was purified using a Milli-Q water purification system (Millipore, Bedford, MA, USA).

### 4.2. Plants Material

The raw material of *G. elegans* plants was cultivated in Longyan City, Fujian Province, China (N 24°43′12″, E 116°43′48″). Vegetative whole plants of *G. elegans* were collected in the area in 2016. Crude samples of *G. elegans* were dried in the sunlight for 2–3 days and milled into a powder. Then, the powder was passed through a 100-mesh sieve to obtain the *G. elegans* powder. Associate Professor Qi Tang at Hunan Agricultural University authenticated the samples. The samples were stored in our laboratory, and the voucher number is 1537201809.

### 4.3. Animal Experiments

Nine male ternary hybrid pigs approximately 60 days old (20 ± 2 kg) were obtained from Hunan New Wellful Co., Ltd. (Changsha, China). Four were fed a complete diet as the control group, and the other five were fed *G. elegans* (a complete diet supplemented with 2% *G. elegans* powder) as the experimental group. After 45 days of continuous feeding, administration of *G. elegans* was stopped, and all pigs were fasted for 12 h [15]. The experimental protocol was performed in accordance with Animal Care and Use of China. Then, all the pigs were slaughtered (before slaughter, blood was collected from the common artery using vacuum blood collection vessels) 6 h after the last feeding. Bile and 11 tissue samples were obtained from each pig, including the heart, liver, lung, spleen, brain, spinal cord, adrenal gland, genitals, thigh muscle, abdominal muscle and back muscle. Instantly, anticoagulated blood was collected and centrifuged at 1200 rpm for 15 min. Since the defecation time of pigs is uncontrollable, all urine and feces excreted within six hours after stopping feeding were collected and stored at −80 °C.

#### 4.3.1. Plasma Samples

Before analysis, pig plasma was warmed to room temperature, and 200 μL of each plasma sample was mixed with 1 mL of 1% formic acid-acetonitrile to precipitate the proteins, followed by centrifugation at 10,000 rpm for 10 min. The supernatant was filtered through a 0.22-μm microbore cellulose membrane for HPLC/QqTOF-MS analysis. Treatment of the plasma samples was based on our previous study [16].

#### 4.3.2. Urine Samples

Urine (200 μL) was added to 1 mL of 1% formic acid-acetonitrile, vortexed 2–3 times for 1–2 min each time and centrifuged at 10,000 rpm for 10 min. The supernatant was filtered through a 0.22-μm microbore cellulose membrane for HPLC/QqTOF-MS analysis. Treatment of the urine samples was similar to the treatment of the plasma samples.

#### 4.3.3. Feces Samples

Two grams of feces were accurately weighed and mixed with 4 mL of 1% formic acid-acetonitrile and then centrifuged at 10,000 rpm for 10 min. One milliliter of supernatant was collected and filtered through a 0.22-μm microbore cellulose membrane for HPLC/QqTOF-MS analysis. Treatment of the fecal samples was similar to the treatment of the plasma samples.

#### 4.3.4. Bile Samples

A 200 μL aliquot of bile was mixed with 1 mL of 1% formic acid-acetonitrile to precipitate the proteins and then centrifuged at 10,000 rpm for 10 min. The supernatant was filtered through a 0.22-μm microbore cellulose membrane for HPLC/QqTOF-MS analysis. The treatment of bile samples was similar to the treatment of the plasma samples. Treatment of the bile samples was based on our previous study.

#### 4.3.5. Tissue Samples

Tissue sample samples (2 g) were accurately weighed and mixed with 4 mL of 1% formic acid-acetonitrile. The samples were vortexed 2–3 times for 1–2 min each time and centrifuged at 10,000 rpm for 10 min. The supernatant was poured into a clean 10.0-mL centrifuge tube, the liquid was dried completely with nitrogen, and then the samples were dissolved in acetonitrile (200 μL) and 0.1% formic acid (800 μL). After all the solids had dissolved, 1 mL of the supernatant was filtered through a 0.22-μm microbore cellulose membrane for HPLC/QqTOF-MS analysis.

#### 4.3.6. HPLC/QqTOF-MS Analysis Conditions

An Agilent 6530 Q-TOF mass spectrometer coupled with an Agilent 1290 HPLC system was used (Agilent Technologies, Palo Alto, CA, USA). The HPLC chromatographic system was equipped with an autosampler, a rapid-resolution binary pump, a vacuum degasser, a thermostatic column compartment and a UV detector. A Thermo-C18 column (2.1 mm × 150 mm i.d.; particle size, 3.5 μm) was used for the separation. The mobile phase consisted of 0.1% formic acid in water (A) and acetonitrile (B). The gradient was as follows: 0–5 min, 10% B; 5–20 min, 10–90% B; 20–25 min, 90% B; and 25–30 min, 10% B. The injection volume was 5 μL, and the flow rate was 0.3 mL/min. The column temperature was maintained at 30 °C.

The mass spectrometer was equipped with an electrospray ionization source and was operated in positive mode. Mass spectrometric analyses were carried out in full-scan MS mode with a mass range of *m*/*z* 50–1000 and auto MS/MS acquisition. The operating parameters were as follows: gas temperature, 300 °C; capillary voltage, 4.0 kV; nebulizer pressure, 35 psi; sheath gas temperature, 350 °C; sheath gas flow rate, 11 L/min; skimmer voltage, 65 V; and fragmentor voltage, 175 V. Nitrogen was used as the nebulizing gas at a flow rate of 9 L/min. Accurate mass measurements of each peak from the total ion chromatograms (TIC) were obtained using an automated calibrant delivery system to provide the mass corrections. The calibration solution contained internal reference masses at *m*/*z* 121.0508 and 922.0098 in positive ion mode. All data acquisition was controlled by Agilent Mass Hunter software (version B.01.03, build 1.3.157.0 2).

## Figures and Tables

**Table 1:** Retention time, formula, observed mass, mass error, fragment ion and biotransformation of twenty natural products and their metabolites in pigs as detected by HPLC/QqTOF-MS.

Compound	RT (min)	Molecular Formula	[M + H]^+^ (*m*/*z*)	Error (ppm)	Fragment Ions (*m*/*z*)	Compound Class	Name	Proposed Metabolism	Source
**H1**	5.0	C_19_H_20_N_2_O_2_	309.1579	6.02	236.1023, 191.0609, 91.0533, 56.0469	Alkaloid, Gelsemine-type	Unknown		B
**H2**	5.2	C_20_H_22_N_2_O_2_	323.1750	1.26	236.1076, 115.0551, 77.0367, 70.0641	Alkaloid, Gelsemine-type	Gelsemine		B
**H6**	13.4	C_20_H_20_N_2_O_3_	337.1565	−5.45	236.1052, 91.0501	Alkaloid, Gelsemine-type	21-Oxogelsemine		B, U
**H6-M1**	6.0	C_20_H_22_N_2_O_3_	339.1667	10.7	206.0973, 77.0389			+2H	B
**H6-M2**	9.8	C_20_H_20_N_2_O_4_	353.1500	−1.18	218.0893, 180.0933, 91.0536, 77.0355			+O	U
**H6-M3**	13.4	C_26_H_28_N_2_O_9_	513.1845	4.41	337.1425, 91.0490, 77.0335			+GlcA	B, U
**H10**	5.4	C_19_H_22_N_2_O_5_	359.1604	−0.7	311.11170, 80.0467, 68.0465	Alkaloid, Gelsedine-type	11,14-Dihydroxygelsenicine		(B), U
**H11**	5.6	C_19_H_22_N_2_O_4_	343.1634	5.36	312.1356, 281.1184, 108.0689, 96.0817, 80.0452	Alkaloid, Gelsedine-type	11-Hydroxygelsenicine		B, U, P
**H11-M1**	2.4	C_25_H_30_N_2_O_10_	519.1978	−0.92	312.1504, 281.1258, 108.0839, 80.0476			+GlcA	B, U
**H12**	6.6	C_19_H_22_N_2_O_4_	343.1640	3.61	312.1428, 80.0491	Alkaloid, Gelsedine-type	14-Hydroxygelsenicine		P
**H14**	9.7	C_19_H_22_N_2_O_3_	327.1703	0.06	265.1240, 108.0703, 95.0869	Alkaloid, Gelsedine-type	Gelsenicine		P
**H15**	10.9	C_21_H_24_N_2_O_5_	385.1758	0	354.1537, 309.1507, 180.0876, 134.0581	Alkaloid, Gelsedine-type	15-Hydroxyhumantenoxenine		B, U
**H16**	11.6	C_19_H_22_N_2_O_4_	343.1631	6.24	238.0790, 128.0493, 118.0639	Alkaloid, Gelsedine-type	Hydroxyl of gelsenicine		B, U
**H18**	11.4	C_20_H_22_N_2_O_5_	371.1571	8.24	340.1368, 295.1354, 120.0412, 91.0508	Alkaloid, Gelsedine-type	Gelsemolenine B		U
**H22**	7.6	C_19_H_20_N_2_O_2_	309.1551	11.53	194.0904, 167.0698, 115.0490	Alkaloid, Sarpagine-type	Oxokoumidine		U
**H23**	7.9	C_19_H_20_N_2_O	293.1650	−0.55	218.0904, 194.0917, 167.0721, 115.0486, 91.0548	Alkaloid, Sarpagine-type	Dehydrokoumidine		B, U, F, P
**H23-M1**	6.3	C_25_H_28_N_2_O_8_	485.1920	−0.33	309.1506, 194.0918, 167.0697, 154.0607, 115.0543			+O + GlcA	U
**H23-M2**	6.5	C_19_H_20_N_2_O_2_	309.1586	3.75	291.1457, 234.1227, 194.0946, 193.0890			+O	Only detected in tissue samples
**H30**	11.5	C_20_H_24_N_2_O_4_	357.1804	1.36	311.1188, 178.1097, 108.0794, 77.0377	Alkaloid, Humantenine-type	14-Hydroxyrankinidine		B, U, F
**H30-M1**	7.3	C_26_H_32_N_2_O_10_	533.2136	−1.18	326.1654, 311.1506, 164.1064, 148.0401,			+GlcA	B, U
**H31**	13.6	C_21_H_26_N_2_O_3_	355.2006	2.88	309.1609, 122.0968	Alkaloid, Humantenine-type	Humantennine		Only detected in tissue samples
**H32**	7.2	C_20_H_22_N_2_O	307.1786	6.17	277.1684, 220.1106, 238.1197, 130.0643, 70.0643	Alkaloid, Koumine-type	Koumine		Only detected in tissue samples
**H37**	4.7	C_16_H_24_O_10_	377.1422	5.38	165.0797, 119.0854, 105.0678, 93.0710	Iridoids	9-Hydroxysemperoside		U
**H39**	4.6	C_10_H_16_O_4_	201.1119	1.18	119.0859, 103.0541, 91.0538, 77.0391	Iridoids	Gelsemiol		U
**H44**	17.5	C_30_H_44_O_3_	453.3410	−10.78	209.1656, 114.0907, 96.0787, 69.0680	Triterpene	3-keto-urs-11-en-13β(28)-olide		B, U, P, F
**H45**	17.7	C_20_H_24_N_2_O_5_	373.1758	−2.15	260.0920, 214.0844, 130.1252	Alkaloid, Gelsedine-type	GS-2(11-Methoxy-14-Hydroxygelsenicine)		Only detected in tissue samples
**H46**	8.3	C_19_H_22_N_2_O	295.1801	1.33	277.1667, 156.0797, 144.0781, 138.0900, 108.0778	Alkaloid, Sarpagine-type	Koumidine		Only detected in tissue samples

Note: “H” means natural products; “M” means metabolites (M1 to M3 means the natural product has three metabolites identified); “U” means urine sample; “P” means plasma sample; “F” means fecal sample; “B” means bile sample; and “()” means low concentration.

**Table 2:** The peak heights of compounds in different tissue samples.

Tissues	Gelsemine (H2)	11-Hydroxygelsenicine (H11)	Gelsenicine (H14)	Dehydrokoumidine (H23)	14-Hydroxyrankinidine (H30)	Humantennine (H31)	Koumine (H32)	3-keto-urs-11-en-13β (28)-olide (H44)	GS-2(11-Methoxy-14-Hydroxygelsenicine) (H45)	Koumidine (H46)	H23-M2	H30-M1
Brain	8 × 10^6^	2.9 × 10^5^	-	0.9 × 10^6^	2.1 × 10^5^	2 × 10^5^	4 × 10^5^	2.9 × 10^7^	2.2 × 10^5^	3.1 × 10^5^	-	-
Spinal cord	3 ×10^5^	-	-	1 × 10^6^	-	-	-	-	2.1 × 10^5^	-	-	-
Heart	8.2 × 10^5^	8 × 10^5^	2.9 × 10^5^	2.9 × 10^6^	6.1 × 10^5^	1.1 × 10^5^	2.6 × 10^5^	3.9 × 10^7^	-	-	-	-
Liver	-	-	-	2.9 × 10^5^	-	-	-	-	-	-	-	-
Lung	2.0 × 10^6^	-	1.9 × 10^5^	3 × 10^7^	2.9 × 10^7^	-	-	-	-	5.9 × 10^6^	3.1 × 10^5^	-
Spleen	2.5 × 10^6^	5.8 × 10^5^	4.9 ×10^5^	6 × 10^6^	1.5 × 10^6^	-	3.2 × 10^5^	-	-	1 × 10^5^	7 × 10^4^	-
Adrenal gland	-	-	-	0.7 × 10^6^	-	-	-	-	-	-	-	-
Testis	6.1 × 10^5^	1.3 × 10^6^	2 × 10^5^	2 × 10^6^	4.2 × 10^5^	1.0 × 10^5^	3.9 × 10^5^	3 × 10^7^	-	-	5.6 × 10^4^	3.7 × 10^5^
Thigh muscle	2.0 × 10^6^	1.4 × 10^6^	3 × 10^5^	4.4 × 10^6^	0.75 × 10^6^	0.9 × 10^5^	0.5 × 10^5^	-	-	-	-	-
Abdominal muscle	1.25 × 10^6^	1 × 10^6^	4 × 10^5^	2.1 × 10^6^	3.9 × 10^5^	-	6 × 10^5^	-	-	-	-	-
Back muscle	7.9 × 10^5^	5 × 10^5^	1.8 × 10^5^	1.9 × 10^6^	3.1 × 10^5^	8 × 10^4^	2.5 × 10^5^	4.1 × 10^7^	-	-	-	-

“-” means that it was not detected in the tissue sample.

## Data Availability

All data generated or analyzed during the present study are included in this published article.

## Sample Availability

Samples of the compounds are not available from the authors.
